# Validation of the Italian Version of the Web Screening Questionnaire for Common Mental Disorders

**DOI:** 10.3390/jcm13041170

**Published:** 2024-02-19

**Authors:** Giada Pietrabissa, Michelle Semonella, Gloria Marchesi, Stefania Mannarini, Gianluca Castelnuovo, Gerhard Andersson, Alessandro Alberto Rossi

**Affiliations:** 1 Dipartimento di Psicologia, Università Cattolica del Sacro Cuore, 20123 Milano, Italy; gloria.marchesi03@icatt.it (G.M.); gianluca.castelnuovo@unicatt.it (G.C.); 2 Clinical Psychology Research Laboratory, IRCCS Istituto Auxologico Italiano, 20149 Milano, Italy; 3 Department of Psychology, Bar-Ilan University, Ramat Gan 52900, Israel; michelle.semonella@biu.ac.il; 4 Department of Philosophy, Sociology, Education, and Applied Psychology, Section of Applied Psychology, University of Padova, 35131 Padova, Italy; stefania.mannarini@unipd.it (S.M.); a.rossi@unipd.it (A.A.R.); 5 Center for Intervention and Research Studies on the Family, University of Padova, 35131 Padova, Italy; 6 Department of Behavioural Science and Learning, Linköping University, 58183 Linköping, Sweden; gerhard.andersson@liu.se; 7 Department of Clinical Neuroscience, Karolinska Institute, 17177 Solna, Sweden

**Keywords:** psychological assessment, online, web-based intervention, anxiety, depression, panic, alcohol problems, obsessive-compulsive disorder, clinical psychology

## Abstract

Background: The ever-increasing spread of Internet-based systems for common mental disorders has generated the need for brief online screening methods. This study aims to test the psychometric properties of the Web Screening Questionnaire (WSQ) to examine its suitability for screening for common mental health problems among a community sample of Italian adults. Methods: A total of 1282 subjects (F = 819; mean age = 42.05) answered the WSQ. Its discriminant characteristics were examined with other validated selected scales for measuring mental health widely used in the Italian population using sensitivity, specificity, and area under the curve (AUC), as well as positive (PPV) and negative predictive values (NPV). Results: Most of the WSQ subscales exhibited moderate to high specificity values. Specifically, the scales of ‘agoraphobia’ (0.947; 95%CI [0.934, 0.960]), ‘anxiety’ (0.959; 95%CI [0.946, 0.970]), and ‘panic disorder’ (0.973; 95%CI [0.964, 0.981]) showed the highest values whilst the ‘obsessive-compulsive’ dimension had the lowest value at 0.838, 95%CI [0.815, 0.861]. With exceptions observed for ‘depression’ (0.716; 95%CI [642, 798]) and ‘alcohol abuse’ (0.760; 95%CI [560, 920]), instead, the WSQ demonstrated critical sensitivity values (<0.6) in all dimensions. Conclusions: The WSQ was appropriate for discriminating between people with and without a psychiatric condition, as it helps to confirm the absence of disorders. However, further diagnostic procedures are required, in case of a positive WSQ screening result.

## 1. Introduction

A growing workforce and mental health services shortage paired with an enormous demand for mental health services has led to increased interest in diverse digital solutions [1]. These include online consultations, mobile applications accessed via smartphones or tablets, and text messaging services designed to improve the psychological health of users, among others. Furthermore, research has shown that Internet-based interventions can be as effective as face-to-face psychotherapy for a variety of psychological issues [2,3,4,5]—such as anxiety [6,7], depression [8], eating disorders [9], and adjustment disorders [10]—not only in controlled trials but also in routine clinical care [11,12].

These interventions can be delivered with minimal or no therapist input [9] and have established cost-effectiveness compared with face-to-face treatment.

The advantages of these programs include the provision of immediate support (i.e., no waiting time), improved access for people who have variable schedules or an important workload, greater flexibility, and removal of many barriers that prevent individuals from accessing face-to-face treatment (e.g., cost, inconvenience, distance, and stigmatization) [13,14].

Given the rise of Internet-based interventions [15,16] for common mental problems [17,18], conducting interviews for routine screening has become even more impractical. However, a precise and reliable diagnosis and measurement are of utmost importance in Internet-based treatments, just like in traditional face-to-face settings [19].

This has generated the need for a reliable and valid online self-rating screening measure for psychiatric disorders that is comprehensible and quick to administer.

In fact, the psychometric characteristics of the instruments used in online surveys have traditionally been established through studies conducted in face-to-face settings, often using pen and paper formats, rather than online administration [19]. But simply translating validated psychological measures from paper to Web format can lead to variations in the results, known as the measurement effect [20], thus affecting the validity [21]. This variation may arise due to differences in participants’ participation, comprehension of questions, and the influence of social desirability bias across different survey administration modes. User experiences based on screen size, hardware, operating system, browser, and internet service provider are other aspects that need to be considered when administering online surveys rather than using a traditional face-to-face format.

Research comparing results obtained using different survey methods (e.g., paper-and-pencil vs. online surveys) on the same sample reported large to small significant differences or even no significant differences [22,23,24,25,26,27]. This indicates the need for further research on this issue, as it is commonly argued that norms must be collected separately for paper and pencil and Internet administration [28].

Therefore, validation studies should be conducted to establish the psychometric properties of psychological measures intended to be used for online surveys. This is particularly true for questionnaires that assess common mental disorders, as they can quickly assist clinicians and researchers in identifying people at risk of having a mental disorder and improving clinical decision making.

The Web Screening Questionnaire (supplementary WSQ) was designed by Donker et al. [29] to quickly identify the most common psychiatric conditions. This online questionnaire comprises only 15 items derived from Marks et al.’s screening questionnaire [30] and takes less than 5 min to complete.

In its original validation study on a Dutch community sample, the WSQ has shown strong validity for disorders like social phobia, panic disorder with agoraphobia, agoraphobia without panic disorder, obsessive-compulsive disorder (OCD), and alcohol abuse or dependence with sensitivity ranging from 0.72 to 1.00 and specificity from 0.63 to 0.80. It also shows moderately good psychometric properties for depressive disorder, generalized anxiety disorder (GAD), post-traumatic stress disorder (PTSD), specific phobia, and panic disorder without agoraphobia with sensitivity ranging from 0.80 to 0.93 and a specificity from 0.44 to 0.51 [29].

Depression, specific phobia and social phobia, PTSD, and alcohol abuse or dependence are assessed by two items, while for the presence of generalized anxiety disorder, panic disorder with agoraphobia, agoraphobia without panic disorder, OCD, and suicidal ideas single items are used for the evaluation.

The elements comprising the depression dimension (*n* = 2) were recovered from the SQ (item #1) and Composite International Diagnostic Interview (CIDI) (item 2), with answer choices on an 8-point Likert scale and Yes/No options, respectively. A single question assessing anxiety (item #3) derived from the Generalized Anxiety Disorder Scale (GAD) and is scored on a 0–3 Likert scale. Panic disorder (item #4) was measured using item #2 of the Panic Disorder Severity Scale—Self-Report (PDSS-SR) on a scale ranging from 0 to 4. A series of yes/no questions from the SQ was used to screen for the presence of agoraphobia (item #5), specific phobia (item #6 and item #7), and social phobia (item #8 and item #9). The elements that compose the dimension of PTSD (*n* = 2) were retrieved from the Mini-international neuropsychiatric interview (MINI) (item #10) and the SQ (item #11), both answered yes or No. A single item (#12) from the Yale–Brown Obsessive-Compulsive Scale (YBOCS) scored on a 5-point Likert scale is representative of the presence of OCD. Alcohol problems were detected through two items from the Alcohol Use Disorders Identification Test (AUDIT), scored on a 0–4 (item #13) and 0–5 Likert scale (item #14). Last, suicidal intention (item #15) was advised using a single item scored on a four-point Likert scale from the SQ. Cut-off scores were set as follow: Depression: item #1 ≥ 5 and item #2 = 1; GAD: item #3 ≥ 2; Panic: item #4 ≥ 1; Panic with Agoraphobia: item #4 ≥ 1 and item #5 = 1; Agoraphobia: item #5 = 1; Specific phobia: item #6 or item #6 ≥ 1; Social phobia: item #8 = 1 and item #9 = 1; PTSD: item #10 = 1 or item #11 = 1; OCD: item #12 ≥ 1; Alcohol Abuse/Dependence: item #13 ≥ 2 and item #14 ≥ 3; and Suicide: item #5 ≥ 3.

Since the WSQ is currently not available for use among the Italian population, the purpose of the present study is to examine its psychometric properties among adults recruited from the general population in Italy.

## 2. Materials and Methods

A cross-sectional research approach was used to examine the validity of the Italian version of the WSQ among adults from the general population.

### 2.1. Translation and Cultural Adaptation

International guidelines [31,32] for translating the WSQ were followed. First, two experienced clinical psychologists independently translated the questionnaire from English to Italian. To maintain consistency, an independent translator performed a back-translation. The final version of the WSQ was tested on a sample of 30 individuals from the general population, to assess the clarity of the items. No additional modifications were required.

### 2.2. Sample Size Determination

The sample size was planned a priori and the following formula [33] was used:

$$N_{minimum}=\frac{Z_{\alpha /2}^{2}\hat{P}(1-\hat{P})}{d^{2}*PREV}$$

where *N_minimum_* is the minimum sample size required to correctly estimate the sensitivity of the WSQ, $Z_{\alpha /2}$ is equal to 1.96, $\hat{P}$ is a pre-determined value of sensitivity that is determined by previous published data, *d* is the margin of error, and *PREV* is the prevalence of the disorder. A sensitivity of 0.80, a margin of error equal to 0.07, and a prevalence of 0.10 were considered [34]. A minimum of 1255 participants were guaranteed.

### 2.3. Participants

The participants were recruited from the general Italian population. The research included individuals who met the following criteria: (1) being over 18 years old, (2) fluent in Italian, and (3) providing their informed consent to participate. Those with visual or cognitive impairments that hindered the completion of the questionnaire were excluded. Participation was voluntary and no financial compensation was provided to the participants.

### 2.4. Procedures

This study was completed solely online and hosted by the questionnaire tool Qualtrics. The recruitment ad included a link placed on the main social networks (i.e., Instagram, Facebook, Twitter). Furthermore, in line with previous studies [35,36,37], personal invitations and advertisements were placed on university campuses, cafés, and libraries in Milan and Padua, Italy.

The initial page contained a detailed description of the study, inclusion, and exclusion criteria along with any potential risks that may occur as a result of participation. The subjects were then asked to acknowledge that they had read the terms and conditions and were aware of any potential risks by signing an online informed consent form.

Following informed consent, participants were asked to complete a survey consisting of 10 sets of questions: demographic questions, the WSQ, the short form of the Post-Traumatic Symptom Questionnaire (PTSQ) [38,39,40], as well as selected dimensions of both the Patients Health Questionnaire (PHQ) and the 90-Revised Symptom Checklist (SCL-90-R).

Upon completion of the survey, participants were provided access to a debriefing page containing information about the study objectives and methodology, along with contact details for support services.

### 2.5. Measures

Demographic information included gender, age, education, civil status, and employment status. The participants were asked to self-report if they had ever been diagnosed with a mental disorder and, in the event of a positive answer, to specify which one(s) (multiple-answer question). Furthermore, participants were asked to self-report if they have ever sought help for a mental health problem and to which professional(s) (multiple answer question), and if they have ever taken or are taking psychotropic drugs (yes/no answer).

In addition, Italian validation of the following self-report measures was administered:

The Patient Health Questionnaire (PHQ) [41,42] evaluates the presence of eight psychological disorders, divided into threshold disorders (those that met specific DSM-IV diagnoses: major depressive disorder, panic disorder, other anxiety disorder, and bulimia nervosa), and subthreshold disorders (those whose criteria encompass fewer symptoms than are required for any specific DSM-IV diagnoses: other depressive disorder, probable alcohol abuse/dependence, somatoform, and binge eating disorder). For this study, the 9-item depression module (known as PHQ-9), the 7-item anxiety module (known as GAD-7), the 7-item panic disorder, and the 5-item alcohol abuse dimension were administered.

The diagnosis of major depression requires the presence of 5 or more of the 9 criteria of depressive symptoms for at least “more than half the days” in the past 2 weeks, with one of the symptoms being depressed mood or anhedonia. Calculating depression severity involves assigning scores of 0, 1, 2, and 3 to the response categories “not at all”, “several days” “more than half the days”, and “nearly every day”, respectively, for each of the nine elements of the PHQ-9. The total PHQ-9 score, ranging from 0 to 27, is derived by summing these assigned scores. Furthermore, the severity of depression is then interpreted based on the total score, with cut-off points established at scores of 5, 10, 15, and 20. Specifically, scores of 5, 10, 15, and 20 signify thresholds for mild, moderate, moderately severe, and severe depression, respectively.

The diagnosis of generalized anxiety requires that you answer more than half of the days to the first item and at least three other items. Calculating anxiety severity using the GAD-7 involves assigning scores of 0, 1, 2, and 3 to the response categories “not at all”, “several days”, “more than half the days”, and “nearly every day”, respectively, for each of the seven items. The total GAD-7 score, ranging from 0 to 21, is obtained by adding these assigned scores. In this context, scores of 5, 10, and 15 serve as cut points for categorizing the severity of anxiety, representing mild, moderate, and severe anxiety, respectively.

Panic disorder is identified if at least 8 of 11 symptoms (Yes/No answer) are present over the past month.

Alcohol problems are detected if at least one of the 5 alcohol-related risk behaviors in the last six months is present during the past 6 months (Yes/No answer).

The 90-Revised Symptom Checklist (SCL-90-R) [43] measures the severity of a wide range of psychological symptoms and is widely used in clinical practice and research. It consists of 90 items scored on a 5-point Likert scale from 0 (not at All) to 4 (Extremely) specifying how much each statement has bothered the respondents over the past 7 days. The SCL-90-R assesses nine scores along the primary symptom dimensions (somatization, obsessive–compulsive, interpersonal sensitivity, depression, anxiety, hostility, phobic anxiety, paranoid ideation, and psychoticism). For the present study, the 10 elements comprising the obsessive-compulsive subscale, the 9 elements creating the interpersonal sensibility subscale, and the 7 elements creating the phobic anxiety subscale were used. According to the literature, a cut-off score equal to 1 (=presence of disorder) was used for all subscales.

The Post-traumatic Symptom Questionnaire (PTSQ) [38,39,40] is a 12-item self-report scale that measures the presence of subjective distress after exposure to traumatic events. The PTSQ comprises three dimensions: intrusion, avoidance, and hyperarousal measured with a Likert-type response format ranging from 1 (not at all) to 5 (extremely) with a cut-off score indicating the absence/presence of post-traumatic stress [38]. For the present study, the brief version (6 items; PTSQ-SF) was used [40].

### 2.6. Statistical Analysis

The presence/absence of depression measured by the WSQ has been related to the presence/absence of depression as measured by PHQ9. The presence/absence of anxiety measured by the WSQ has been related to the presence/absence of anxiety as measured by GAD7. The presence/absence of social phobia as measured by the WSQ has been related to the presence/absence of anxiety as measured by the dimension of interpersonal sensibility of the SCL-90-R. The presence/absence of panic disorder, panic with agoraphobia, and agoraphobia as measured by the WSQ has been related to the presence/absence of panic disorder, panic with agoraphobia, and agoraphobia as measured by the panic disorder module of the PHQ. The presence/absence of specific phobia as measured by the WSQ has been related to the presence/absence of specific phobia as measured by the phobic anxiety dimension of the SCL-90-R. The presence/absence of OCD as measured by the WSQ has been related to the presence/absence of OCD as measured by the obsessive-compulsive subscale of the SCL-90-R. The presence/absence of PTSD as measured by the WSQ has been related to the presence/absence of PTSD as measured by the phobic anxiety dimension of the SCL-90-R. The presence/absence of OCD as measured by the WSQ has been related to the presence/absence of OCD as measured by the PTSQ-SF. The presence/absence of alcohol abuse/dependence as measured by the WSQ has been related to the presence/absence of alcohol abuse/dependence as measured by the alcohol abuse dimension of the PHQ.

The general accuracy-validity of the WSQ was assessed with the area under the ROC curve (AUC; 5000 stratified bootstrap resamples). Swets’ benchmarks were used to interpret the AUC [44,45,46]: AUC = 0.50, null; AUC from 0.51 to 0.70, small; AUC from 0.71 to 0.90, moderate; AUC from 0.91 to 0.99, large; and AUC = 1.00, perfect accuracy. Furthermore, the sensitivity (SEN), specificity (SPE), and accuracy (ACC) of each subscale of the WSQ were tested against the corresponding disorder. Specifically, SEN is the probability that a person with a disorder is positive at the screening, while SPE is the probability that a person without a disorder is negative at the screening. ACC is the ability of a test to correctly classify subjects (true positives and true negatives) against a criterion. Additionally, positive predicted values ((PPV) probability of a positive diagnosis after positive screening) and negative predicted values ((NPV) probability of a negative diagnosis after negative screening) were also calculated.

Lastly, to determine the strength of the difference between those who scored positive on the WSQ and those who screened negative, a series of t tests for each screening instrument were performed separately. Specifically, the diagnosis obtained on the WSQ was used as the independent variable, while the score obtained on the questionnaire corresponding to the disorder of interest was used as the dependent variable. The strength of the difference was interpreted using Hedge’s g and its benchmarks [47]: null (<0.20), small (from 0.20 to 0.49), moderate (0.50 to 0.79), and large (>0.80). Also, the separation index (1 − *η*) index was used to quantify the magnitude of a difference between groups [48,49] — also with non-normal distributions and of samples with different sizes. The separation index is the opposite of the overlapping index (that is, the 1-overlapping index): it ranges from 0 (=perfect overlap) to 1 (=perfect separation) and should be interpreted as a normalized effect size [48].

## 3. Results

### 3.1. Sample Characteristics

The sample of this study was made up of 1282 participants: 463 men (36.1%) and 819 females (63.7%), in the age range of 18 to 82 years (mean = 42.05, SD = 14.317). The descriptive statistics are reported in Table 1.

### 3.2. Concordance between the Screening Questionnaires and the Web Screening Questionnaire (WSQ)

**Figure 1:**
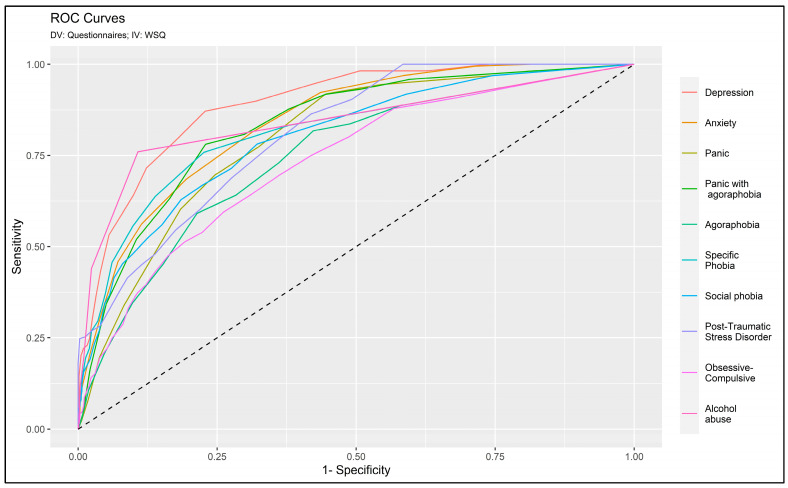
ROC curves.

Table 2 delineates the concordance between each diagnosis categorized according to the screening questionnaires (i.e., PHQ9, GAD7, etc.) and the WSQ. All AUC and ACC values were deemed good, as illustrated in Figure 1. In detail, the lowest discriminating subscale was ‘obsessive-compulsive’ (AUC = 0.732; ACC = 0.736) followed by ‘agoraphobia’ (AUC = 0.746; ACC = 0.858) ‘social phobia’ (AUC = 0.799; ACC = 0.805) and ‘panic disorder’ (AUC = 0.799; ACC = 0.848). Most of the scales showed both AUC and ACC values greater than 0.80 such as ‘PTSD’ (AUC = 0.810; ACC = 0.819), ‘specific phobia’ (AUC = 0.811; ACC = 0.894), ‘panic with agoraphobia’ (AUC = 0.832; ACC = 0.914) and ‘anxiety’ (AUC = 0.840; ACC = 0.861). The two most discriminating scales were ‘alcohol abuse’ (AUC = 0.841; ACC = 0.902) and ‘depression’ (AUC = 0.891; ACC = 0.863).

On the one hand, sensitivity showed critical values (<0.6) for most disorders with exceptions noted for ‘depression’ (0.716) and ‘alcohol abuse’ (0.760). However, the specificity exhibited moderate to high values for all subscales. In fact, the lowest value was 0.838 for the ‘obsessive-compulsive’ scale and the highest values were 0.947, 0.959, and 0.973, respectively, for ‘agoraphobia’, ‘anxiety’, and ‘panic disorder’. It should be noted that the other scales showed a specificity greater than 0.84.

### 3.3. Assessing the Strength of Differences

**Figure 2:**
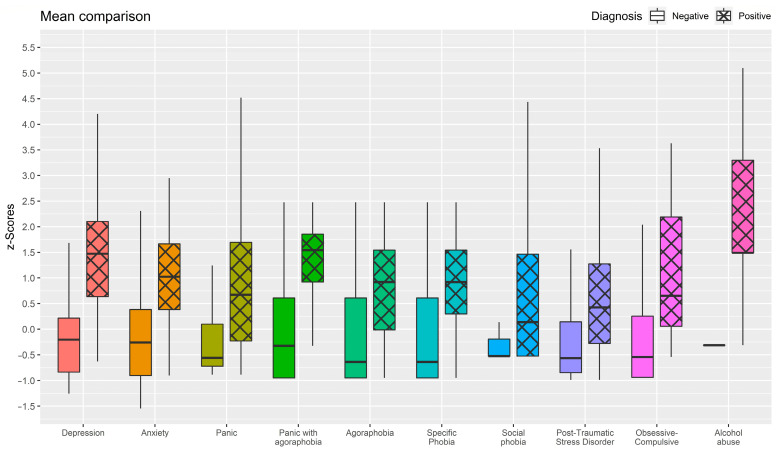
Boxplot representing differences in mean scores on the screening questionnaires between subjects who have received a diagnosis through the WSQ (positive) and those who have not.

Table 3 and Figure 2 showed that respondents who voted yes for a specific WSQ “diagnosis” showed markedly higher means (*p* < 0.001) on the associated validation questionnaire in comparison to those who voted no for the same WSQ “diagnosis”. Moreover, all Hedges’ *g* were higher than 0.9 suggesting a strong difference between the two groups. Accordingly, the separation index (1 − *η*) suggests that the two WSQ diagnosis-related groups exhibit a notable separation.

## 4. Discussion

This study evaluated the psychometric properties of the WSQ to detect the presence of mood, anxiety, obsessive-compulsive, post-traumatic stress disorders, and alcohol abuse among a community sample of Italian adults.

### 4.1. Principal Findings

Overall, the WSQ was successful in discriminating between individuals with and without a psychiatric disorder. On average, response of subjects who tested positive for the diagnosis of mental disorders in WSQ consistently showed higher values compared with those without WSQ diagnosis. For example, individuals with depression exhibited markedly elevated mean scores compared with those without a depression diagnosis measured by the WSQ, with an extremely high effect size. Similarly, mean WSQ scores for anxiety were significantly higher among those presenting this psychiatric condition compared who did not test positive for anxiety problems at the WSQ with significantly substantial impact magnitude.

Respondents with PTSD also showed significantly higher mean WSQ post-traumatic stress scores than those without diagnosis of PTSD according to the WSQ and high effect size.

Furthermore, those who presented alcohol abuse disorder according to the WSQ showed moderately higher mean values than those who did not test positive for this disorder according to the WSQ criteria, with an increased effect size.

A similar trend was observed for the remaining phobic-related problems, namely social phobia, panic, panic with agoraphobia, agoraphobia, specific phobia, and obsessive-compulsive: subjects who tested positive for the diagnosis through the WSQ consistently exhibited significantly higher means scored in these dimensions compared with respondents without WSQ diagnosis.

This indicates that the WSQ can distinguish between people with or without mental disorders so powerfully that those with a diagnosis appear to be markedly pathological.

In addition, the findings on the sensitivity, specificity, and AUC values suggest that the WSQ has some advisable screening characteristics.

Its high specificity suggests that it may help to confirm the absence (true negative) of all psychiatric conditions, meaning that it cannot rule out the disorders. This is especially important when people who are identified as having a condition should undergo a more detailed screening for the presence and intensity of mental disorders.

However, the low sensitivity for most of the disorders measured by the WSQ was somewhat surprising, given the higher values reported in a previous sample, which demonstrate both the high sensitivity and high specificity of the tool [29]. The sensitivity reported in another research was similar to that found in this study [50].

The difference could in part be due to the sample and the measuring methods. In fact, in this study, we only employed self-report screening questionnaires and did not conduct clinical diagnostic interviews to assess the presence of a current psychiatric diagnosis [29,50]. Another possible explanation for the low sensitivity of the WSQ could lie in the proposed cut-off criteria.

### 4.2. Strengths and Limitations

To our knowledge, this is the only valid measure of common psychiatric conditions available in Italy for planning and conducting an online survey—the WSQ.

A strength of this research is the extensive inclusion of participants, constituting a well-balanced and representative sample.

Another crucial aspect of this study is the use of rigorous statistical methods to test hypotheses and identify patterns and relationships in the data, leading to increased objectivity and credibility in the results and drawing meaningful conclusions that can inform the work in the field. To this end, only psychometric instruments validated in Italian were used to inform the results.

However, the generality of this finding is limited to Italian-speaking respondents who are Internet-literate. Furthermore, the sensitivity and specificity of the Italian version may not apply to clinical samples. Therefore, the present results need to be confirmed in other populations.

Furthermore, the low prevalence of disorders in respondents devalues the predictive value of the tool, in addition to the fact that the clinical value of self-reported diagnosis remains debatable.

Lastly, since no clinical interviews were conducted to confirm (or disconfirm) the diagnosis made by self-report tools, and the results of this work are based solely on the single validation study available in the literature [29], it was not possible to explore, adjust, and propose alternative cutoffs for the use of the WSQ in the Italian population. This would have improved the screening capacity and accuracy of the tool; therefore, its diagnostic performance for clinical and research purposes in both clinical and non-clinical samples.

Future studies should parallel the WSQ with a validated clinical semi-structured interview to gather more in-depth information on participant attitudes, thoughts, and actions and generate confirmatory results on the absence of mood, anxiety, obsessive-compulsive, post-traumatic stress disorders, and alcohol abuse among respondents.

Despite its limitations, the WSQ remains a valuable and rapid online screening tool (it takes about two/three minutes to complete) for common mental disorders.

It offers an accessible and convenient means of assessing mental health conditions, particularly in non-clinical samples who may not have easy access to traditional mental health services.

The WSQ can be easily disseminated through online platforms, reaching a wide range of individuals regardless of their geographic location or socio-economic status.

This population-level approach allows identification of trends and patterns in mental health within communities, facilitating the development of targeted prevention and intervention strategies [51,52] to address identified individual risk factors.

This targeted approach ensures that healthcare resources are directed towards those who need them the most, thereby improving efficiency within the healthcare system.

The provision of valuable insights into the prevalence and characteristics of mental health problems within non-clinical samples can information public health policies and initiatives aimed at promoting mental well-being and preventing mental illness at a population level.

## 5. Conclusions

The WSQ functions as a concise and easy to administer survey designed to identify conditions such as depression, GAD, panic disorder (with or without agoraphobia), social phobia, specific phobia, OCD, PTSD, and alcohol abuse or dependence. Its validity in the Italian general population has been established by comparing it to the standardized self-repost measure of mental illnesses. The findings suggest that the WSQ could potentially serve as a cost-effective and brief online screening tool for common psychiatric conditions, particularly in primary care settings. In fact, it could aid healthcare providers in pre-consultation screening, with positive screens prompting more thorough diagnostic evaluations. By facilitating early detection, personalized intervention thought telehealth services, and population-level screening the WSQ has the potential to improve diagnostic accuracy and reduce the healthcare expenditures.

## Figures and Tables

**Table 1:** Sample descriptive statistics.

	*n*	%
Civil status (*n*, %)		
Single	246	19.2%
In a relationship	421	32.8%
Married	508	39.6%
Separated	41	3.2%
Divorced	50	3.9%
Widowed	16	1.2%
Education (*n*, %)		
Middle school degree	74	5.8%
Professional qualification	70	5.58%
High school degree	491	38.38%
Bachelor degree	167	13.08%
Master degree	367	28.68%
Ph.D.	113	8.88%
Job status (*n*, %)		
Student	148	11.5%
Employee worker	657	51.2%
Freelance worker	227	17.7%
Unemployed	45	3.5%
Housewife	72	5.6%
Retired	56	4.4%
Other	77	6.0%
Diagnosed mental health disorder (*n*, %)		
None	693	54.1%
Anxiety	416	32.4%
Obsessive-compulsive disorder	30	2.3%
Depression	157	12.2%
EDs	107	8.3%
Sexual Disorders	19	1.5%
Post-traumatic stress disorder (PTSD)	82	6.4%
Substance-use related disorders (SRAD)	8	0.6%
Specific phobia	11	0.9%
Personality disorders	16	1.2%
Psychosis	5	0.4%
Other	21	1.6%
Mental health consultation (*n*, %)		
None	682	53.2%
Psychiatrist	140	29.6%
Psychologist	380	29.6%
Psychotherapist	236	18.4%
Other professional	20	1.6%
Psychiatric drug (*n*, %)		
Yes	105	8.2%
No	1177	91.8%

Notes: *n* = number of individuals; % = percentage.

**Table 2:** Agreement between the screening questionnaires and the WSQ.

				χ^2^	AUC	ACC	SPE	SEN	NPV	PPV
		PHQ9 Diagnosis								
Depressive disorder		No	Yes							
WSQ-depression	No	1029	144	240.68 *	0.891	0.863	0.877	0.716	0.971	0.351
	Yes	31	78		[0.862, 0.919]	[0.843, 0.882]	[0.858, 0.895]	[0.624, 0.798]	[0.962, 0.979]	[0.306, 0.397]
		GAD7 Diagnosis								
Generalized anxiety disorder		No	Yes							
WSQ-anxiety	No	1043	45	154.32 *	0.840	0.861	0.959	0.314	0.887	0.575
	Yes	133	61		[0.814, 0.867]	[0.847, 0.875]	[0.946, 0.970]	[0.253, 0.376]	[0.878, 0.896]	[0.490, 0.663]
		SCL-90-R INT SENS								
Social phobia		No	Yes							
WSQ-Social Phobia	No	898	128	197.83 *	0.799	0.805	0.875	0.523	0.880	0.511
	Yes	122	134		[0.769, 0.830]	[0.785, 0.824]	[0.855, 0.895]	[0.465, 0.586]	[0.867, 0.895]	[0.464, 0.562]
		Panic Diagnosis								
Panic disorder		No	Yes							
WSQ-Panic	No	1020	28	184.13 *	0.799	0.848	0.973	0.286	0.859	0.705
	Yes	167	67		[0.770, 0.827]	[0.839, 0.858]	[0.964, 0.981]	[0.251, 0.315]	[0.854, 0.864]	[0.558, 0.852]
		Panic Diagnosis								
Panic with agoraphobia		No	Yes							
WSQ-Panic and Agoraph.	No	1143	66	112.88 *	0.832	0.914	0.945	0.397	0.963	0.305
	Yes	44	29		[0.787, 0.877]	[0.902, 0.928]	[0.939, 0.967]	[0.302, 0.492]	[0.957, 0.969]	[0.227, 0.383]
		Panic Diagnosis								
Agoraphobia		No	Yes							
WSQ-Agoraphobia	No	1064	59	58.865 *	0.746	0.858	0.947	0.226	0.896	0.379
	Yes	123	36		[0.706, 0.786]	[0.845, 0.871]	[0.934, 0.960]	[0.163, 0.289]	[0.888, 0.904]	[0.287, 0.471]
		SCL-90-R PHOB ANX								
Specific phobia		No	Yes							
WSQ-Specific Phobia	No	1106	27	154.19 *	0.811	0.894	0.976	0.268	0.910	0.597
	Yes	109	40		[0.772, 0.851]	[0.882, 0.906]	[0.967, 0.984]	[0.201, 0.342]	[0.903, 0.918]	[0.487, 0.707]
		SCL-90-R OCD								
Obsessive-compulsive disorder		No	Yes							
WSQ-OCD	No	771	149	134.09 *	0.732	0.736	0.838	0.475	0.802	0.536
	Yes	190	172		[0.702, 0.762]	[0.713, 0.757]	[0.815, 0.861]	[0.420, 0.528]	[0.785, 0.819]	[0.490, 0.581]
		PTSQ-SF Diagnosis								
Post-traumatic stress disorder		No	Yes							
WSQ-PTSD	No	961	123	134.54 *	0.810	0.819	0.887	0.449	0.898	0.420
	Yes	109	89		[0.781, 0.838]	[0.800, 0.839]	[0.868, 0.905]	[0.384, 0.520]	[0.887, 0.910]	[0.367, 0.477]
		Alcohol Diagnosis								
Alcohol abuse/dependence		No	Yes							
WSQ-Alcohol	No	1138	119	106.14 *	0.841	0.902	0.905	0.760	0.995	0.138
	Yes	6	19		[0.752, 0.931]	[0.884, 0.920]	[0.886, 0.924]	[0.560, 0.920]	[0.990, 0.998]	[0.108, 0.168]

Notes: * *p* < 0.001; all of the χ^2^ (chi-square test) have 1 degree of freedom (*df*), 95%CI [Lower; Upper]. AUC = Area under the curve; ACC = accuracy; SPE = specificity; SEN = sensibility; NPV = negative predicted value; PPV = positive predicted value. PHQ9 = Patient health questionnaire 9 (depression); GAD-7 = Generalized anxiety scale; SCL-90-R INT SENS = SCL-90-R Interpersonal sensibility scale; Panic Diagnosis = panic module of the Patient health questionnaire; SCL-90-R PHOB ANX = SCL-90-R phobic anxiety subscale; SCL-90-R OCD = SCL-90-R obsessive-compulsive subscale; PTSQ-SF = Post-Traumatic Symptom Questionnaire—short form; Alcohol Diagnosis = alcohol module of the Patient health questionnaire.

**Table 3:** Differences in mean scores on the screening questionnaires between subjects who have received a diagnosis through the WSQ and those who have not.

	Diagnosis on WSQ		Statistics			
	No	Yes				
	M (SD)	M (SD)	*t*	*p*-value	g	1 − *η*
Depression (range 0–27)	5.278 (3.986)	13.505 (5.870)	−14.328	<0.001	1.97	0.591
Anxiety (range 0–21)	4.210 (2.777)	8.170 (2.755)	−18.317	<0.001	1.43	0.471
Social Phobia (range 0–4)	0.442 (0.520)	1.237 (0.847)	−14.367	<0.001	1.33	0.435
Panic (range 0–11)	2.407 (2.932)	5.889 (2.847)	−16.509	<0.001	1.19	0.384
Panic with Agoraphobia (range 0–11)	2.806 (3.080)	6.973 (2.764)	−12.425	<0.001	1.36	0.468
Agoraphobia (range 0–11)	2.674 (3.036)	5.648 (3.214)	−11.474	<0.001	0.97	0.324
Specific phobia (range 0–4)	0.106 (0.240)	0.382 (0.558)	−10.895	<0.001	1.61	0.615
Obsessive-compulsive (range 0–4)	0.531 (0.580)	1.122 (0.819)	−12.537	<0.001	0.90	0.330
Post-Traumatic Stress (range 6–36)	9.758 (4.132)	15.980 (6.184)	−13.612	<0.001	1.38	0.365
Alcohol abuse (range 0–5)	0.146 (0.494)	1.520 (1.295)	−5.296	<0.001	2.64	0.732

Depression = Patient health questionnaire 9 (PHQ9); Anxiety = Generalized anxiety scale (GAD-7); Social phobia = SCL-90-R Interpersonal sensibility scale; Panic = panic module of the Patient health questionnaire; Panic with agoraphobia = panic module of the Patient health questionnaire; Agoraphobia = panic module of the Patient health questionnaire; Specific phobia = SCL-90-R phobic anxiety subscale; Obsessive-compulsive = SCL-90-R obsessive-compulsive subscale; Post-Traumatic Stress = Post-Traumatic Symptom Questionnaire—short form; Alcohol abuse = alcohol module of the Patient health questionnaire.

## Data Availability

The data presented in this study are available on request from the corresponding author.

## Supplementary Materials

The following supporting information can be downloaded at: https://www.mdpi.com/article/10.3390/jcm13041170/s1, The Web Screening Questionnaire (WSQ)–Italian version.
