# Supplementary material for: Validation of the Italian Version of the Web Screening Questionnaire for Common Mental Disorders
Source: J Clin Med. 2024 Feb 19;13(4):1170. doi: 10.3390/jcm13041170 (PMC10889998; doi:10.3390/jcm13041170)
Supplement: Supplementary file 1 [file jcm-13-01170-s001.zip › jcm-2825097-supplementary.pdf]

WEB SCREENING QUESTIONNAIRE (WSQ)

1. **Cerchia un numero dalla scala sottostante per mostrare quanto sei preoccupato dal sentirti infelice o depresso:**

Quasi per niente		Leggermente preoccupato		Decisamente preoccupato		Fortemente preoccupato		Estremamente preoccupato
0	1	2	3	4	5	6	7	8

2. **Provi perdita di interesse e/o piacere nella maggior parte delle cose, come lavoro, hobby e altre cose che solitamente ti piacciono?**

☐ SI ☐ NO

3. **Nelle ultime due settimane, quante volte sei stato infastidito dal non riuscire a rilassarti?**

Per nulla	Diversi giorni	Più della metà dei giorni	Quasi ogni giorno
0	1	2	3

4. Un attacco di panico è un improvviso impeto di paura o disagio accompagnato da almeno 4 dei sintomi elencati di seguito che devono raggiungere il loro picco entro 10 minuti. I sintomi sono: battito cardiaco accelerato, sudorazione, tremore, mancanza di respiro, sensazione di soffocamento, dolore/fastidio al petto, nausea, vertigini/ senso di debolezza, sensazione di irrealtà, intorpidimento/formicolio, brividi o vampate di calore, paura di perdere il controllo o di impazzire, paura di morire.

Se hai avuto attacchi di panico durante la scorsa settimana, quanto sono stati angoscianti (disturbanti, spaventosi) mentre stavano accadendo? Se non hai avuto attacchi di panico ma solo alcuni sintomi, rispondi con riferimento a essi.

Per nulla angoscianti/ spaventosi *(distressing),* o nessun attacco di panico o sintomi limitati durante la scorsa settimana	Leggermente angoscianti/ spaventosi (attacchi non troppo intensi)	Moderatamente angoscianti/ spaventosi (attacchi intensi ma gestibili)	Fortemente angoscianti/ spaventosi (attacchi molto intensi)	Estremamente angoscianti/ spaventosi (estrema angoscia durante tutti gli attacchi)
0	1	2	3	4

5. **Eviti i luoghi pubblici da cui ti sembra difficile scappare o li sopporti con evidente sofferenza o ansia? (es. trasporti pubblici, negozi/centri città, code, cinema, edifici sconosciuti, luogo distanti da casa).**

☐ SI ☐ NO

6. **Sei estremamente ansioso o eviti oggetti o situazioni specifiche?**

☐ SI ☐ NO

7. **Hai paura di: animali (es. Cani, ragni, serpenti, gatti, uccelli, topi, insetti) o condizioni mediche (es. sangue, dentista, iniezione, chirurgia, ospedale, dottore) o situazioni specifiche (es. Autobus, negozio affollato, tunnel ascensore, aereo, ponte o guida in auto)**

☐ SI ☐ NO

8. **Hai evitato situazioni sociali per paura che l'attenzione potesse essere su di te??**

☐ SI ☐ NO

9. **Hai paura o ti imbarazza essere osservato, stare al centro dell'attenzione o hai paura di essere umiliato? (incluse situazioni come parlare in pubblico, mangiare in pubblico con altri, scrivere mentre qualcuno guarda o trovarsi in situazioni sociali).**

☐ SI ☐ NO

10. I tuoi sintomi sono iniziati dopo aver vissuto, assistito o dovuto affrontare un evento estremamente traumatico come la morte reale o la minaccia di morte o lesioni gravi a te o a qualcun altro? (ad es. incidente grave, aggressione sessuale o fisica, attacco terroristico, presa in ostaggio, rapimento, rapina, incendio, morte inaspettata, guerra, disastro naturale ...)

☐ SI ☐ NO

11. Hai mai vissuto un evento traumatico?

☐ SI ☐ NO

12. Le ossessioni sono pensieri, impulsi o immagini ricorrenti che sono indesiderati, sgradevoli, inappropriati, invadenti o angoscianti (ad esempio l'idea di ferire i tuoi figli anche se sai che non lo faresti mai). **Quanto tempo hai dedicato alle ossessioni nell'ultima settimana?**

0 ore/giorno o nessuna ossessione	0-1 ore/giorno	1-3 ore/giorno	3-8 ore/giorno	>8 ore/giorno
0	1	2	3	4

13. Quante bevande alcoliche assumi in una tipica giornata in cui bevi?

Nessuna	1-2	3-4	5-6	7-9	10 o più
0	1	2	3	4	5

14. Quanto spesso bevi sei o più bevande alcoliche in un'occasione?

Nessuna	Meno di una volta al mese	Una volta al mese	Una volta a settimana	Tutti i giorni o quasi
0	1	2	3	4

15. Di recente ti è venuta in mente l'idea di farti del male o di toglierti la vita?

Mai	Mi è passato per la mente ma non lo farei	Ci ho pensato seriamente ma mi sono fermato	Lo farei se ne avessi l’opportunità
0	1	2	3
